# The culture of care within psychiatric services: tackling inequalities and improving clinical and organisational capabilities

**DOI:** 10.1186/1747-5341-7-12

**Published:** 2012-09-28

**Authors:** Micol Ascoli, Andrea Palinski, John Arianda Owiti, Bertine De Jongh, Kamaldeep S Bhui

**Affiliations:** 1 Tower Hamlets Cultural Consultation Service, Queen Mary, University of London, Old Anatomy Building, Charterhouse Square, London, EC1M 6BQ, UK; 2 Cultural Consultation Service & Wolfson Institute of Preventive Medicine, Queen Mary, University of London, Old Anatomy Building, Charterhouse Square, London, EC1M 6BQ, UK

**Keywords:** Cultural consultation, Cultural formulation, Ethnography, Institutional racism, Culture and mental health

## Abstract

**Introduction:**

Cultural Consultation is a clinical process that emerged from anthropological critiques of mental healthcare. It includes attention to therapeutic communication, research observations and research methods that capture cultural practices and narratives in mental healthcare. This essay describes the work of a Cultural Consultation Service (ToCCS) that improves service user outcomes by offering cultural consultation to mental health practitioners. The setting is a psychiatric service with complex and challenging work located in an ethnically diverse inner city urban area. Following a period of 18 months of cultural consultation, we gather the dominant narratives that emerged during our evaluation of our service.

**Results:**

These narratives highlight how culture is conceptualized and acted upon in the day-to-day practices of individual health and social care professionals, specialist psychiatric teams and in care systems. The findings reveal common narratives and themes about culture, ethnicity, race and their perceived place and meaningfulness in clinical care. These narratives express underlying assumptions and covert rules for managing, and sometimes negating, dilemmas and difficulties when considering “culture” in the presentation and expression of mental distress. The narratives reveal an overall “culture of understanding cultural issues” and specific “cultures of care”. These emerged as necessary foci of intervention to improve service user outcomes.

**Conclusion:**

Understanding the cultures of care showed that clinical and managerial over-structuring of care prioritises organisational proficiency, but it leads to inflexibility. Consequently, the care provided is less personalised and less accommodating of cultural issues, therefore, professionals are unable to see or consider cultural influences in recovery.

## Introduction

The need to include the cultural dimension in healthcare delivery is widely recognised. In multi-cultural, multi-ethnic and multi-faith societies, there are persistent ethnic inequalities in the use of health services; for example, there are significant differences in access to services, experience and outcomes between the majority population and patients from black and ethnic minority background [1-6]. The tone of the UK debate on how to reduce these inequalities fluctuates between polemics about the injustices of ethnic inequalities as a driver for change and protests about the uselessness of politically correct efforts to eradicate inequalities; the latter then undermine policies and actions that are potential remedies to ethnic inequalities in service users’ experiences and outcomes. The recent debate in the UK on institutional racism in mental health care and how to tackle it is an excellent example; different levels and paths of analysis can lead to paralysis and inertia [7-9]. Another example is the call for improved cultural competence or capability for all practitioners working within mainstream services on the one hand [10], and failing this, a call for culture/ethnic specific services, on the other [11]. Neither position is acceptable if resources are limited or if these suggestions are seen as politically correct propositions that are unnecessary, a position that is now adopted by the current UK government. Whatever the political climate, at an academic level the limitations of the inflexibility of the Western psychiatric classification and clinical methodology when applied to different cultures and the need to include the cultural dimension in clinical care have been highlighted for several years [12].

### Cultural consultation & anthropology

Debates about policy and politics may seem remote from the daily realities of providing clinical assessment, diagnosis and treatment. Cultural consultation services feature among the solutions to improve care practices and reduce inequalities. This approach is informed by anthropological methods and concepts and has gathered momentum precisely because it focuses far more on cultural influences within the clinical encounter [13,14]. An evaluation of the Canadian model of Cultural Consultation found evidence of improved diagnostic accuracy, culturally relevant care planning and workforce satisfaction, and of the need for healthcare professionals to receive further training in working with interpreters [15]. As such, cultural consultation is a clinical process, and given its reliance on anthropological research methods, specifically ethnography, it is also an evaluative tool that helps understand the cultures of health care settings [16-21].

A number of mixed-methods evaluations of public services now use anthropological methods which have even been used more directly to understand conflict, for example, on inpatient wards and in the context of terrorism [22-24]. In this paper we report on a cultural consultation service working in a mental health service, specifically with specialist teams such as home treatment, assertive outreach, community mental health, and early intervention. This paper outlines its implementation, the service model and the narratives of care that emerged during our evaluation of the service.

## Methodology

### Aims & objectives

The Tower Hamlets Cultural Consultation Service (ToCCS) was designed and commissioned to improve the delivery of culturally capable mental health care by mainstream services catering for a culturally diverse urban population of East London. The ToCCS worked alongside five secondary services as a tertiary resource and was based on the Cultural Consultation approach designed in Canada [15]. The ToCCS was designed as an adaptation of the Canadian model that took into account some relevant characteristics of the UK context and its health service, such as the specialist teams that deliver care, the types of ethnic and cultural diversity of staff working in the specialist mental health teams in inner London, the local systems of commissioning and the UK policy context. The ToCCS therefore worked at three different levels of services: clinical cultural consultation, workforce development and organisational consultancy. The educational and change management approach adopted a developmental, iterative and non-linear process that we expected would improve the quality of clinical care and outcomes over time; we expected to replicate the findings from Canada and to evaluate additional impacts at the organisational and commissioning level, whilst revealing more of the hidden processes in healthcare delivery that are subsumed under the term of organisational culture. The work was part of an NHS commissioned service, and all the data were audit or evaluation information sought with the consent of any participating patients. Given this was an NHS commissioned service audit and evaluation project, formal ethical approval was not sought.

### The structure & processes of ToCCS

The ToCCS staff, called “cultural consultants” included a clinical psychiatrist, a forensic trained mental health nurse, a medical anthropologist, a part time administrator, and an outcomes manager. The team skills included mental health nursing, psychiatry, psychotherapy, counseling, research and medical anthropology. The cultural consultants established ongoing relationships with the specialist mental health teams for eighteen months (assertive outreach, home treatment, community mental health team, early intervention) as well as with individual clinicians, managers and commissioners. ToCCS consultants participated in and observed ‘usual care’ practices such as teams meetings and received referrals from these. Referrals also came from carers, service users, commissioners and managers. Ethnographic methodology, especially direct and participant observation, allowed the cultural consultants to collect narratives.

After each referral, the ToCCS team discussed the narratives of the referring team, the appropriateness of the referral and orientated the cultural consultant towards a meaningful formulation of the initial request. The cultural consultant then met the referrer to better explore the desired outcomes and to access the clinical documentation on the patient, then fed back the findings to the rest of the ToCCS team. Once a referral was understood to potentially benefit from clinical cultural consultation, a continuing process of in-depth analysis of the referral and the observable care processes helped to identify common themes and narratives about culture of the referring team as well as of individual patients. The ToCCS consultant undertook ongoing evaluations of what motivated the referral, as well as the difficulties encountered in care, by the use of documentary analysis, and consultation with relevant experts. This culminated in a comprehensive report to the referrer. The reports included a synthesis of the key observations, narratives, and the ToCCS recommendations based on multidisciplinary peer supervision within the team. These reports and other field notes and documents together formed the body of evidence that formed the basis of the evaluation of our work that now is included in the analysis in this paper.

### Data collection & analysis

In accord with the principles of cultural consultation service models, the evaluation methods are also largely drawn from anthropology. Ethnography is a qualitative research method developed by anthropologists as a way of gathering rich and thick descriptions of how people live their lives and what stories they tell themselves; the narratives and observations captured by ethnography reveal what constitutes culture and cultural practices [25]. Thus anthropologists gather and interpret field notes, recordings (written or typed or electronic clinical records and reports) and information gathered from conversations and observations of routines and rituals; together these expose what constitutes ordinary cultural practices. The underlying assumptions and power relations are inferred from verbal and non-verbal communications, and validity of such inferences relies on the depth of the information and its consistent and repeated presence. This method of participant and non-participant observations lets the anthropologist discover the rules by which people actually live their lives rather than only taking account of what people say they do. The approach is a powerful method that has been applied to organisational analysis and development, as well as to individual clinical encounters in healthcare settings [26,27]. Due to its focus on lived experiences, an ethnographic approach has the potential to improve both quality of care and outcomes. Thus anthropological methods seek highly valid data for a particular setting and context, rather than widely generalisable data, although the methods permit the discovery of new theories and approaches that can be tested more generally.

In this paper, we report on a synthesis of different types of data: a) key consistent narratives found across all teams and for which there was sufficient evidence for us to conclude that saturation was reached in the emergent information; we judged saturation to be reached if we felt further referrals, contacts, work and iterations of our analysis would be unlikely to offer any more fresh or new insights; b) there were also numerous other themes and narratives unique to specific referrals and teams, but these informed the overall body of evidence about how cultural consultation works, for whom it works and for which sorts of problems; c) we were also able to discern where there were unexpected challenges and these themselves constitute important information, the narratives of which are also presented. Narrative data was subject to thematic analysis for commonly recurring themes [28]. Through this process, we searched for, and located themes or patterns, through group consensus. This occurred as we organized and described narrative data in detail, thus creating links among themes as we understood them and subjected them to critical interpretation. This led to an insightful analysis of organizational and teams’ cultures, and to the understanding of “cultures” within teams.

The thematic categories by which these narratives are reported reflect their natural grouping as they emerged during the consultation process. During the project, the emergent categories of the narratives were modified to accommodate new information, but where new information was not easily accommodated, a separate thematic category was introduced. Using this approach, the findings grouped into: a) narratives about the primary reason for referral; b) narratives about what constitutes culture and therefore cultural competence and cultural capability; c) narratives revealing an overall culture of care.

## Results and discussion

### Narratives of primary reasons for referrals

Explicit, overt motivations for referrals to the team were grouped and are illustrated in Table 1.

**Table 1:** Narratives of primary reasons for referrals

Perplexing and complex clinical presentations, lack of sufficient knowledge or understanding about the cases	
·	Diagnostic clarification or confirmation, or request for second opinion
·	Identification of possible cultural factors influencing an unclear clinical presentation
·	Exploration and clarification of previous traumatic history, migration history and possible impact on current presentation
Lack of engagement or progress and failed treatment alliance:	
·	Exploration of possible cultural barriers to engagement and treatment adherence for non-collaborative patients
·	Request for mediation between family and services to improve treatment alliance and outcomes
·	Exploration of cultural factors influencing family dynamics and difference between culturally appropriate family structures and pathological family constellations
Exploration and resolution of cultural conflicts	
·	Exploration of conflict between sexual orientation and culture of origin
·	Exploration of and guidance for resolution of conflict between culture of origin and UK culture
·	Resolution of intergenerational conflict between immigrant patients and step children
Racism and discrimination	
·	Exploration of patients’ experience of racism and discrimination and evaluation of possible impact on current symptoms and presentation
Defensive practices	
·	Requests for generic involvement in care planning alongside other agencies for particularly risky cases
Information requests	
·	Requests for information on culturally appropriate community links and resources
·	Requests for information on migration agencies and procedures, or international organisations

Some of these overt requests can be grouped under the general heading of receiving help with cultural complexity and identifying alternative and creative ways of working with service users to improve their experiences and outcomes. An in-depth analysis of referrals and care processes, however, revealed some deep seated narratives about cultural capability and an overall culture of care, as outlined below.

### Emergent narratives on cultural capability and implications for understanding the culture of culture

We identified four main covert themes around culture and cultural capability. Below, we illustrate each theme through relevant case studies.

### A confusion of identity and negation of culture, ethnicity and race

ToCCS had several meetings with the teams it would work alongside, starting with initial presentations about the service. The reaction of some staff was guarded and there was limited engagement with the subject. Some staff questioned the remit of ToCCS and asked what practically ToCCS provided that they could not already do, and how ToCCS treated culture in any different way than their teams already did. One manager was interested in knowing how ToCCS was maintaining funding in the current financial climate, when other services were being cut.

A manager asked for the ToCCS consultants to attend the team meetings (of two hours duration) and to take part in case discussions. However discussion around culture was not forthcoming and the cultural consultant was instead asked for information about third sector services. It was for the cultural consultant to take the floor and prompt the team’s reflections on culture when the opportunity arose. This seemed necessary but it did distract from in-depth work with individual members of staff along with the service users. On several occasions, when individual members of staff were approached for potential referrals, they appeared uncomfortable and reluctant to refer patients.

Some members of staff reacted as they perceived that the ToCCS posed a threat to their team’s existence given recent discussion about sustainable NHS services and the need for savings of up to 20%. A member of staff asked whether the ToCCS considered support workers obsolete, perhaps showing who was under threat but also misunderstanding that the ToCCS was not a support workers’ service.

There were several suggestions put forward for how ToCCS should work. Some staff suggested that the ToCCS’ presence could be similar to how they worked with child protection, or that the working relationships could mirror that of non-statutory service organisations like Work Connect, or that ToCCS could be used as a Drug and Alcohol Service: anything it seemed other than focus on culture, race and ethnicity. One member of staff who could not see any reason for making referrals to the ToCCS stated that “if they encountered an Eskimo the ToCCS could possibly be of some help”.

Some staff never made any referrals. Some contacted us, appropriately, for information on third sector services, or international organisations for migration and refugees. On one occasion, after a series of clinical presentations aimed at illustrating the complexity of working clinically across cultures, some staff felt criticised and reacted in defence with anger that such matters were being discussed openly.

Although it is difficult to make any firm conclusions, clearly there were some assumptions about what the service offered and what was to be risked by engaging with such a service, as well as little expectation of benefits. The situation improved with time, but such instances were revealing about the predominant assumptions about the role of the cultures of the practitioners, the teams, and the service users in the provision of care. These statements showed early judgements on relative value and worth, and perhaps anxieties about scrutiny of existing practice in the current context of financial constraint and services cuts.

The ToCCS presented their approach, style of work, engagement strategies through presentations, participation, and gathering information and narratives from diverse perspectives. Yet it remained difficult for some staff to grasp the purpose of ToCCS, not by some subtle confusion of roles, but by quite remarkable margins of misunderstanding. For example, a staff member said that she had already done ‘motivational interviewing’ and did not need further training, showing complete negation of all the previous presentations and conversations. Another stated that their existing team was already culturally diverse enough to be able to deal with the cultural dimension appropriately and without any specialist help; different team members were from different ethnic groups and therefore there was no need to consider these issues, as if these would, de facto, be addressed in diagnosis and clinical treatment.

These examples illustrate how raising the issue of culture and cultural capability in clinical care can result in powerful reactions of fear, rejection, mistrust and hostility. These were surprising to us at the time, as our role was to complement rather than replace, however, these sentiments seemed to be explained by additional data presented below highlighting the pressures under which staff were working and the risk averse cultures of everyday practice. A specific issue of importance emerged in narratives of staff, especially minority staff, about their work, their position within the organisation, its hierarchy and its culture.

### Pain, enslavement and suffering of staff

The following narratives emerged showing how much staff struggle with their own safety, role and identity in the work place.

"“Once, on the ward, a service user tried to hit me. I was just in time to avoid getting punched. I went to my manager. She told me: “Well, what can you do, it’s part of the job, at least you did not get injured; be more careful next time”. That’s when I understood they did not care about me”. (nurse)"

"“I’m not going to take the stress of the work. I finish my job and that’s it. I completely unplug. When I go home, I can’t even remember the name of the patients.” (nurse)"

"“Patients, colleagues, managers can abuse me, bully me, enslave me, but only during working hours. After that, that’s it, that’s done. That’s how I keep my sanity.” (nurse)"

"“Where else can I go? There are not so many jobs out there, and in my country it’s even worse.” (nurse)"

"“People in this country are two-faced. In the meetings, they talk, and talk, and talk, then they ask me if I have anything to say. How does that matter, when all decisions have been made already?” (nurse)"

"“How come you don’t work agency? It’s easy money and you’ve got no responsibility” (nurse)"

These narratives, some of which collected from black and ethnic minority staff, clearly illustrate what some of them experience at work in terms of burn-out, depersonalisation, loss of empathy, fatigue, isolation and alienation from a supportive culture. This context for some, can trigger emotions, memories and themes related to the collective legacy of unjust organisations, stigma and discrimination in society as well as in mental health care. The sentiments and outrage may also be informed by historical accounts of injustice, for example, slavery or holocaust, and by the personal stories of staff and the cultures of their upbringing [29]. These feelings around dehumanising events from history may have special salience for specific cultural, religious or ethnic groups, and such feelings can be triggered by contemporary experiences of disempowerment or perceived discrimination. The functions of psychiatric practice to provide care, and yet to contain risk and emotional distress whilst doing so through structures such as detention, restraint, medication, locked wards etc., may actually present staff with an impossible dilemma. Such sentiments, however latent, can be triggered by contemporary contexts which appear to resonate with and re-create oppressive environments. One wonders how such an overwhelmingly structured work environment and organisational milieu could ever be conducive to an attitude of openness, curiosity and willingness to engage in a discourse about patient centred care, personal narratives, and about the role of race, culture and ethnicity in healthcare. An absence of such discussion is likely to impact detrimentally on the diagnosis and treatment options [14].

### Culture is an attribute only of ethnic minorities and patients and not the majority or the organisation

No member of staff raised the issue of how their own culture affected the therapeutic relationship with a patient from a different cultural background. The question of how to bridge the cultural gap between patient and care coordinator was always asked with emphasis on the patient’s culture. Of the 99 referrals received over a one year period, only two were for white British patients. None of the teams referred itself for organisational cultural consultancy, analysis of the team’s culture, conflict mediation or for receiving support to improve intercultural communication among diverse staff.

The ToCCS' work revealed assumptions in staff narratives about cultural competence. First, that this concept only relates to clinical work with ethnic minority patients; second, that the clinicians’ or the organisation’s cultures do not matter and need not be analysed; third, that the clinicians are “culture free”: an unexpected finding for us, given the cultural diversity of the staff in the teams we worked with (however, this perhaps reflects why staff whose cultural lives are a key marker of their distinct identity felt alienated or isolated or badly treated); fourth, that the patient’s culture was the locus of pathology and the place where all the complexity lay, rather than the systems of care and professional practice showing complexities that need to be understood.

### Denial of disability through racial/cultural camouflage

Although work around race, culture and ethnicity often emphasises over treatment or inappropriate diagnoses, the following account shows that this negation of race, culture and ethnicity can lead to under-treatment. The passive acceptance of the patient’s narrative on race, culture and ethnicity lead to the incapacity to recognise disability. Culture, race and the racial discourse were therefore used to justify an ideology of therapeutic nihilism and lack of intervention.

A middle aged man of Caribbean origin with mental illness has refused any kind of engagement with services and treatment for years. He lives in a constant state of severe self-neglect and total social isolation. There is no electricity, heating or hot water in his flat. ToCCS gets involved in his care upon request by the treating team to get in touch with a black consultant psychiatrist, as the patient refuses to see his white European consultant. The referral is accepted, yet the scope of the work is broadened by ToCCS and through fifteen doorstep assessment by the cultural consultant and the care coordinator a more in depth process becomes possible and the clinical cultural consultation process is followed. The consultation process leads to the recognition of a broad narrative of the patient about racism and discrimination, whereby he reaffirms his refusal to engage with any suggested worker on the grounds of race, ethnicity, religious belief, gender, age and professional seniority or orientation, claiming that his needs as a middle aged male of Caribbean descent cannot be met or understood on these various grounds and labelling each attempt at intervention as racist, oppressive, discriminatory and custodialistic. The treating team finds itself trapped and paralysed as the majority of staff accepts this narrative and therefore their potential interventions become identified with discrimination and oppression, leading to therapeutic impasse. The team’s dominant narrative is that the patient is “high functioning” and that his presentation is suggestive of a life-style based on free personal choice, rather than pathology. The cultural formulation clarifies that the patient, far from being articulated and high functioning, is displaying mannerism as found in psychosis: his narrative, although apparently well articulated, is in reality repetitive, stereotyped, poor in content and used to distance the other as a paychological defensive mechanism. The consultation report recommends that the patient is treated under the Mental Health Act and transferred to a rehabilitation unit, to regain the skills he will need in order to live as healthily and as autonomously as possible.

In summary, the analysis of staff narratives about culture and cultural capability beyond the level of the overt referrals included a widespread notion that when it comes to cultural competence or capability, only the patients’ culture matters as it is the locus of all pathology. The idea that only minority patients have a culture is widespread and deeply seated in the practice, as shown by the selection of the cases for referrals, while teams, staff and the organisation are conceptualised as culture-free or culturally neutral. The one exception of this was the belief that ethnic matching is sufficient in itself for the provision of quality services to ethnic minorities. This seemed to be the rationale for some to pursue a deeper level of disengagement from cultural factors on the assumption it was addressed in its totality. So the culture of the professional and the organisation was seen as not relevant or totally addressed with no scope for its consideration in the care processes. We hypothesised that those staff members who totally refused to engage with the ToCCS as they belonged to the same cultural background of their patients did not only consider themselves automatically culturally capable, but this was also a psychological mechanism to avoid their own culture being considered or investigated or being identified with pathology. This makes sense given the risk of having their culture defined as the locus of pathology located in patients. This is in line with the notion that the need for cultural consultation provided by a specialist service is useful only for a minority of patients from “exotic” or “unusual” cultural backgrounds or with “exotic presentations” that are bizarre and fall outside even psychiatric nosology. So culture is conceptualised as an attribute of the alien and accepted as a subject of investigation only when it can be situated at a safe distance from the professional. Narratives about staff cultural and racial discomfort within the organisation tied in with a diffusely guarded reaction of mistrust towards ToCCS to maintain the status quo of existing services. In contrast to specialists interested in cultural psychiatry, in our experience many clinicians are not yet ready to look deeply into their cultural biography and to truly engage in a process of personal and professional development towards the achievement of cultural capability. The identification of the clinicians’ cultural barriers to delivering high quality and equitable services still remains a step too far, or perhaps too demanding personally and professionally.

## Conclusions

We worked with five teams that are part of a wider system, and with a variety of teams and clinicians from a broad range of professional backgrounds, cultures and ethnicities and different levels of seniority. Therefore we believe that our findings are of relevance to other mental health services in urban settings. A clinical approach strictly based on diagnostic coding, evidence-based interventions, adherence to practice guidelines and an excessive degree of risk aversion may limit the range of the interventions that can potentially be offered or acceptable to patients of non-western cultural backgrounds. The current financial climate in the UK and the necessity for the services to meet the targets might result in an excessive pressure on managers and clinicians to divert their attention to the quantitative, rather than the qualitative, aspects of care and service provision, potentially to the detriment of the delivery of personalised, tailored, culturally capable care. In times of financial constraint one of the dominant objectives of the services becomes meeting the set targets to justify their own existence, creating a paradoxical and anti-therapeutic reversal of the patient-clinician relationship, whereby it is the service that needs the patient to survive. The community teams we worked with experienced a climate of uncertainty and continuous redefinition of their remit, structure, staffing levels, targets and budgets, due to the current financial situation and the cuts that are affecting the NHS. The overall context within which the teams operated did not promote an attitude of interest, curiosity and desire to learn how to deal with the cultural dimension in the clinical setting, on many levels and for different reasons. In depth engagement with the cultural dimension of care will largely depend on the clinicians’ willingness to venture in this field. This is in turn a function of their personal biographies and their own conceptualisations of culture, ethnicity and race.

We found that teams’ culture of care is the place where the gap between the high aspirations of the policy level and the users’ frustration at the slow pace of change lies. Ultimately, teams’ cultures of care need to change if we want to achieve high quality of care for patients, better outcomes and improved experience. However, we found a culture of incurableness, as a widespread and deeply seated perspective in the practice of mental health professionals, and this was hard to shift. We suggest that the timeline for this shift might be long. We argue that, in this sense, the community can be seen as the modern “virtual asylum” where incurableness and chronicity take place in post de-institutionalisation psychiatry, not in the fabric of buildings but in the practices of individual clinicians and organised care systems.

In such a context, it is unlikely that the clinical methodology of the cultural consultation, as widespread as it might become in the future, can translate into direct improvement in patients’ experience and outcomes until the culture of culture and the culture of care is tackled alongside this. A tertiary service of cultural consultation might be a privileged point of observation of teams’ cultures, functions and dysfunctions, to clarify where the problems lie, rather than to offer simplistic solutions shaped as a set of clear cut recommendations. Cultural consultation can therefore be also conceptualised, at this stage, as a tool in organisational anthropology more apt to analyse the scale of the problems rather than quickly indicate what the solutions might be.

## Competing interests

The author declares that they have no competing interest.

## Authors’ contributions

KB designed the ToCCS. KB, AP and JAO established relationships with the teams we worked with and presented the service in various academic and clinical forums. AP, JO and MA carried out the clinical work (assessments, cultural formulation, cultural consultation reports) and the ethnographies on which the findings are based. All authors took part in the intake of referrals, clinical meetings, training events, report writing, peer supervision. BDJ carried out the data analysis for the purpose of the service evaluation. All authors conceived of the study, participated in its design and coordination and helped to draft the manuscript. KB provided the theoretical and methodological background to the paper and structured its content. All authors read, revised consecutive drafts, and approved the final manuscript.

## Authors’ information

MA, MD (It) is a consultant psychiatrist and psychotherapist and Honorary Senior Clinical Lecturer at Queen Mary University of London and East London NHS Foundation Trust.

AP, MSc is a medical anthropologist and team manager of the Cultural Consultation Service.

JAO, PhD RMN is a senior mental health nurse, a medical anthropologist, and Honorary Practice Innovation Nurse, East London NHS Foundation Trust.

BDJ, MSc is data analyst at Queen Mary University of London.

KB, MD FRCPsych, is Professor of Cultural Psychiatry & Epidemiology at Queen Mary University of London, and Hon Consultant Psychiatrist, East London Foundation Trust.
